# Phylogeography and population genetics of pine butterflies: Sky islands increase genetic divergence

**DOI:** 10.1002/ece3.5793

**Published:** 2019-11-07

**Authors:** Dale A. Halbritter, Caroline G. Storer, Akito Y. Kawahara, Jaret C. Daniels

**Affiliations:** ^1^ Entomology and Nematology Department University of Florida Gainesville FL USA; ^2^ McGuire Center for Lepidoptera and Biodiversity Florida Museum of Natural History University of Florida Gainesville FL USA; ^3^ Present address: USDA‐ARS Invasive Plant Research Laboratory 3225 College Ave Fort Lauderdale FL 33314 USA

**Keywords:** ddRADSeq, *Neophasia*, phylogeny, Pieridae, *Pinus*, population

## Abstract

The sky islands of southeastern Arizona (AZ) mark a major transition zone between tropical and temperate biota and are considered a neglected biodiversity hotspot. Dispersal ability and host plant specificity are thought to impact the history and diversity of insect populations across the sky islands. We aimed to investigate the population structure and phylogeography of two pine‐feeding pierid butterflies, the pine white (*Neophasia menapia*) and the Mexican pine white (*Neophasia terlooii*), restricted to these “islands” at this transition zone. Given their dependence on pines as the larval hosts, we hypothesized that habitat connectivity affects population structure and is at least in part responsible for their allopatry. We sampled DNA from freshly collected butterflies from 17 sites in the sky islands and adjacent high‐elevation habitats and sequenced these samples using ddRADSeq. Up to 15,399 SNPs were discovered and analyzed in population genetic and phylogenetic contexts with Stacks and pyRAD pipelines. Low genetic differentiation in *N. menapia* suggests that it is panmictic. Conversely, there is strong evidence for population structure within *N. terlooii*. Each sky island likely contains a population of *N. terlooii*, and clustering is hierarchical, with populations on proximal mountains being more related to each other. The *N. menapia* habitat, which is largely contiguous, facilitates panmixia, while the *N. terlooii* habitat, restricted to the higher elevations on each sky island, creates distinct population structure. Phylogenetic results corroborate those from population genetic analyses. The historical climate‐driven fluxes in forest habitat connectivity have implications for understanding the biodiversity of fragmented habitats.

## Graphical abstract

The high‐throughput sequencing approach of ddRADSeq was used to investigate the phylogeography and population structure of two closely related butterfly species restricted to mountain habitats. Despite their flight capabilities and the proximity between some mountain ranges (56 km apart), there was distinct population structure among butterflies in the sky islands of southeastern Arizona. Butterflies in the largely connected belt of pine forest from east–central Arizona northwestward to the Kaibab Plateau exhibited panmixia.

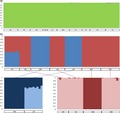

## 1 INTRODUCTION

**Figure 1:**
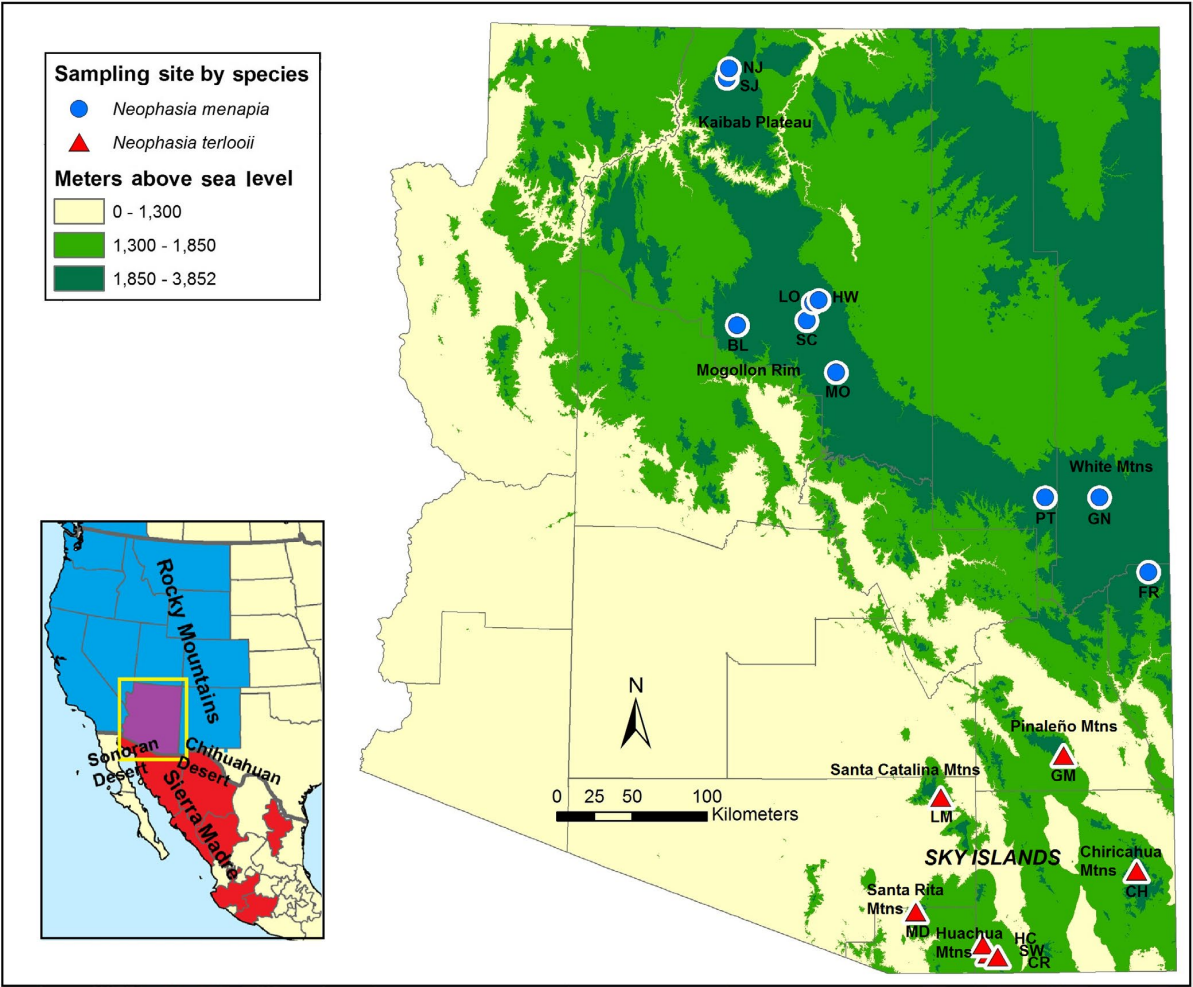
Map of Arizona, showing sampling sites and their two‐letter identifiers. Elevation data were acquired from the National Resources Conservation Service Geospatial Data Gateway. The 1,300–1,850 m elevation range represents a scenario 18,000 C^14^ years before present when the open conifer woodlands occurred in that range, evidenced by packrat middens containing plant matter from open conifer woodlands (Table 3 of Thompson and Anderson (2000)). Elevations above 1,850 m represent the present day start of open conifer woodlands. Inset map shows major ecoregions in black text and shades the state/province‐scale distributions (except for Texas, which has only one record in the extreme SW corner) of each *Neophasia* spp.: blue for *Neophasia menapia* and red for *Neophasia terlooii*. Both species occur in Arizona, and it is therefore shaded purple

The isolated mountain habitats of western North America provide a natural laboratory for investigating the evolutionary processes at work in taxa restricted to naturally or anthropogenically fragmented habitats. The Madrean Archipelago (sky islands) is a region of isolated mountain ranges spanning extreme southeastern Arizona, USA (AZ), southwestern New Mexico, USA, and northern Mexico. The lower elevations (<1,000 m asl) make up the Sonoran Desert to the west and the Chihuahuan Desert to the east. The higher elevations (>2,000 m asl) are interspersed in the gap between the Rocky Mountains to the north and the Sierra Madre Occidental to the south (Figure 1). The arid desert lowlands can serve as a barrier to dispersal for high‐elevation animal species (Holycross & Douglas, 2007; Masta, 2000; Ober, Matthews, Ferrieri, & Kuhn, 2011; Tennessen & Zamudio, 2008). The sky islands are also a region of high species turnover, as dozens of insect genera and hundreds to thousands of insect species reach the northern or southern limits of their ranges there (Felger & Wilson, 1994). As a result of the complex topography, mixing of temperate and tropical climate zones, and convergence of two major mountain ranges, the sky islands are one of the most biologically diverse ecoregions in the world (Skroch, 2008). By studying abundant and ecologically diverse taxa such as insects, we can gain a better understanding of diversification and community structure (Moore et al., 2013).

*Neophasia* butterflies are an excellent system for investigating how the sky islands have shaped species history. These butterflies are coniferous forest specialists and their evolutionary history likely follows that of their forest habitats, which have been hypothesized to fluctuate in area and connectivity in association with climate changes (Thompson & Anderson, 2000). The near‐contact zone of ranges of the two *Neophasia* species meets at the major transition of ecoregions marked by the sky islands in AZ. *Neophasia menapia* (Felder & Felder, 1859) ranges from southwestern British Columbia to the Mogollon Rim and White Mountains of AZ (Scott, 1986), and southeastward through New Mexico to Guadalupe Mountains National Park in Texas (Lotts & Naberhaus, 2016). *Neophasia menapia* has four geographically defined subspecies (Pelham, 2008) and *N. m. magnamenapia* is the subspecies in AZ on which this study was focused (henceforth *N. menapia*). *Neophasia terlooii* (Behr, 1869) is present in the sky islands of southeastern AZ southward to the Sierra Madre Occidental in central Mexico (Bailowitz & Brock, 1991). Both *N. menapia* and *N. terlooii* are exemplary representatives of the two major ecoregions that meet at the sky islands: *N. menapia* from the Rocky Mountain ecoregion and *N. terlooii* from the Madrean ecoregion. Additionally, because *Neophasia* are locally abundant, easy to spot, collect, and identify, and are fairly weak flyers, they are reliably available for collection in sufficient numbers at multiple sites. Additionally, due to their dependence on climatologically sensitive pine forests, they constitute an excellent model taxon to investigate how climate‐induced changes in habitat connectivity influence diversification.

The larval hosts of *N. menapia* include *Abies*, *Pseudotsuga*, *Tsuga*, *Picea*, and *Pinus*, (Cole, 1971; Evenden, 1926; Robinson, Ackery, Kitching, Beccaloni, & Hernández, 2002; Scott, 1986) and *N. terlooii* feeds only on the latter two genera (Arizona Game & Fish Department, 2001). *Pinus ponderosa* (Engelmann), a larval host for both *Neophasia* species, occurs in the more fragmented Madrean evergreen woodlands and is a predominant member of the comparably larger and more contiguous Rocky Mountain coniferous forests (Brown, 1994). There is some overlap in the voltinism of these two species in AZ; *N. menapia* is univoltine, adults are active from mid‐July through August, and *N. terlooii* is bivoltine and is active in low numbers from June through early September with peak adult activity in October. For both species, eggs are laid on live pine needles. Some populations of *N. menapia* in Montana have occasionally undergone population irruptions (Dewey, Ciesla, & Meyer, 1974), but in most cases *Neophasia* are locally abundant and not believed to disperse far from forested habitats containing their host trees.

Given the close and abrupt range disjunction in *Neophasia*, our main objective was to determine whether *Neophasia* species residing in forested habitats of different ecoregions of the sky islands and surrounding area have similar concordant population structure. Using newly discovered SNPs from ddRADSeq libraries generated for this study, we infer with multiple lines of evidence the population structure and phylogeographic history of *Neophasia*. We hypothesized that, despite their capability of flight, genetic structuring of *Neophasia* populations would reflect the degree of current habitat connectivity. We discuss the limits of *Neophasia* dispersal with respect to the geographic layout of their mountain habitats in Arizona. In addition to physiological constraints and climate differences between habitats (Halbritter, Teets, Williams, & Daniels, 2018), the desert and grassland matrices separating the two species' mountain habitats are likely to be dispersal barriers contributing to allopatry.

## 2 MATERIALS AND METHODS

### 2.1 Specimens

*Neophasia menapia* (*n* = 88) and *N. terlooii* (*n* = 69) were collected from 10 and seven field sites, respectively, throughout AZ in the summer and fall of 2013 and 2014 (Figure 1, Table 1). Specimens were kept alive until they were stored at −15°C for a minimum of 12 hr. Frozen butterflies were then placed into 95% ethanol stored at the same temperature until they were moved to the University of Florida, Gainesville, FL, USA, at which point they were stored at −80°C.

**Table 1:** Number of butterflies per sampling site for the ddRADSeq analyses, with ten being the target number

Species	Site	*n*	Species	Site	*n*
*Neophasia menapia* a	BL	10	*Neophasia terlooii*	CH	10
	FR	10		CR	10
	GN	10		GM	10
	HW	3		HC	9
	LO	6		LM	10
	MO	8		MD	10
	NJ	9		SW	10
	PT	6			
	SC	10			
	SJ	10			

a Eighty‐eight *N. menapia* were initially collected, but six were excluded after data filtering.

### 2.2 Library preparation

Thoracic muscle tissue, roughly 3 mm × 1 mm, was removed from each adult butterfly for DNA extraction. Genomic DNA was extracted from tissues using the OmniPrep extraction kit (G‐Biosciences; Cat. # 786‐136) following the manufacturer's protocol, with some adjustments to optimize DNA recovery (Appendix S1). Each DNA extract was quantified using a Qubit dsDNA BR Assay Kit (Life Technologies, Co.; Lot # 1681328). Extractions that contained a DNA concentration of at least 10 ng/µl were used for library preparation.

Library preparation for ddRADSeq followed that of Peterson, Weber, Kay, Fisher, and Hoekstra (2012), with some added quality control measures (Appendix S1). We used the restriction enzymes EcoRI (NEB, R3101 20,000 units/ml) and MseI (NEB, R0525 10,000 units/ml). These two enzymes have been successfully used to obtain 20,737 SNPs in an independent population genomics study on *N. menapia* (Bell, 2012). Unique barcodes, ranging from 8 to 14 bases in length, and adaptors were then ligated to the EcoR1 ends of digestion fragments and common adaptors were ligated to the MseI termini. Each barcoded individual was PCR amplified using Illumina sequencing primers to confirm the success of digestion and ligation reactions. Individuals were then pooled into a single sequencing library. Enzymes and buffers were removed from the pooled library using the QIAquick PCR Purification Kit (Qiagen; Cat. # 28106), fractioned, and fractions between 275 and 475 bases were collected using the Pippin ELF (Sage Science) platform. Illumina flowcell binding sequences were incorporated into the desired fractionation product using PCR, primers were removed using AMPure beads (Beckman Coulter Life Sciences), and lastly single‐end DNA sequencing was performed on the Illumina NextSeq500 platform using a midoutput flowcell for 150 cycles at the University of Florida Interdisciplinary Center for Biotechnology Research.

### 2.3 Raw data processing

Raw sequences containing the multiplexed reads (10.7 GB total) were first assessed for quality by generating FastQC reports (fastqc 0.11.4; Andrews, 2010). Any reads that did not have at least 90% of bases with a Phred score of ≥20 were discarded using FastX quality trimmer from the FASTX_Toolkit (http://hannonlab.cshl.edu/fastx_toolkit/). Filtered sequences were then demultiplexed using FastX_toolkit FASTQ Barcode splitter and either the 14‐, 10‐, 9‐, or 8‐base barcodes. FastQC reports, filtering, and demultiplexing were all performed using the UF instance of Galaxy (Afgan et al., 2018).

Barcodes and the 6‐base pair enzyme cut site were trimmed from the 5′ end and 5–10 bases were trimmed from the 3′ end of the reads from each individual. Bases were trimmed from the 3′ end because the last 5–10 bases tended to have lower Phred scores. Reads were all trimmed to be at most 125 bases in length to facilitate downstream analyses in Stacks. Trimming was accomplished using FASTX_trimmer from the fastx_toolkit. Because we used two restriction enzymes, some of the MseI cut sites were less than 125 bases from the EcoRI cut site at the 5′ end. These shorter reads were removed using FASTX _clipper to yield the final library of demultiplexed files for each individual.

### 2.4 Discovering loci and calling genotypes

We used stacks 1.35 (Catchen, Hohenlohe, Bassham, Amores, & Cresko, 2013) for locus discovery and genotyping. Because a reference genome was not available to assemble loci when processing the reads, we ran the stacks program denovo_map.pl to assemble loci. Unless otherwise specified with a population map, stacks assumes all barcoded individuals belong to a single population. To avoid investigator bias during SNP identification, we did not specify a population map because we did not know if our sampling sites could be considered true populations. Key parameters were set within the range to optimize the discovery of loci and SNPs: ‐m 3 ‐M 3 ‐n 2 ‐T 6 ‐t ‐S ‐b 1 (Mastretta‐Yanes et al., 2015). To produce genepop files and generate summary statistics, the stacks outputs from denovo_map.pl were run through the populations component of stacks with the following parameters: ‐b 1 ‐k ‐p 1 ‐m 7 ‐r 0.80 ‐f p_value ‐t 8 ‐‐structure ‐‐write_single_snp ‐‐genepop ‐‐fasta. The populations component of stacks was run on each species separately to maximize the number of loci retained within each species. This was accomplished by specifying population maps that included only *N. menapia* as one population or *N. terlooii* as one population. For both species, we omitted from downstream analyses any individuals with <60% of genotypes present. This was a conservative filtering step given that Mastretta‐Yanes et al. (2015) used a cutoff of 50%. The stacks populations pipeline was then run again under the same parameters but with the filtered datasets. After such filtering, we retained 82 *N. menapia* and 69 *N. terlooii* (Table 1).

### 2.5 Quantifying genetic variability and spatial relationships

For all downstream analyses with the stacks populations outputs, R 3.2.2 was used (R Core Team, 2015). Expected heterozygosity was computed using function *Hs* from “adegenet” (Jombart, 2008) and deviations from Hardy–Weinberg equilibrium were tested for using the *hw.test* function from “pegas” (Paradis, 2010). We did not know if our sampling sites represented true populations; therefore, we first quantified genetic differentiation by comparing fixation indices (*F*_ST_) between sampling locations within each species. To determine between location fixation indices, we ran *pairwise.fst* from “heirfstat” (Goudat & Jombart, 2015) for populations within each species and computed *F*_ST_ confidence intervals using *boot.ppfst*. Our sampling was spatially clustered with respect to site; therefore, *F*_ST_ comparisons were appropriate (Manel, Schwartz, Luikart, & Taberlet, 2003). A test for isolation by distance was run for each species using *mantel.randtest* from “ade4” (Dray & Dufour, 2007). An isolation by distance plot was generated, and *F*_ST_ values were used to plot genetic distance as a function of geographic distance.

An analysis of molecular variance (AMOVA) was performed on the stacks‐generated genotypes for both species using *poppr.amova* from “poppr” 2.1.1 (Kamvar, Tabima, & Grünwald, 2014). Strata were specified such that each individual was placed in a sampling region (mountain range) and then a site within each region (e.g., there were three sampling sites within the Huachuca Mountains). To test the significance of variations within individuals, between individuals, between sites (i.e., collection sites), and between mountains (i.e., mountain ranges or regions containing one or more sites), *randtest* from “ade4” was used on the AMOVA objects.

### 2.6 Identifying population clusters

To identify genetic clusters and estimate cluster membership probabilities of each individual, we used structure 2.3.4 (Pritchard, Stephens, & Donnelly, 2000) to estimate *K*, searching from *K* = 1–15 with 10 replicates per *K* and a burnin of 100,000 and 150,000 reps after burnin for each replicate. Prior population or location information was not used for inferring clusters and admixture was allowed. Results were interpreted using Structure Harvester (Earl & vonHoldt, 2012) and plots generated using Structure Plot (Ramasamy, Ramasamy, Bindroo, & Naik, 2014). Substructure was investigated in *N. terlooii* for sampling sites in which all individuals had at or near 100% probability assignment to one of two clusters (i.e., minimal admixture). Two datasets, each including only minimally admixed sampling sites from one of the clusters, were analyzed again with structure using the aforementioned parameters, except that only *K* = 1–5 were tested.

### 2.7 Phylogenetic analysis

To further investigate genetic structure, we ran the ddRADSeq data through the pyrad pipeline to infer phylogenetic relationships. Eaton (2014) developed pyrad to maximize phylogenetic information across more distantly related samples from RADseq data. stacks and pyrad differ in that pyrad uses a global alignment algorithm that allows indels. Although the stacks pipeline has been used to generate species‐level phylogenies (e.g., Jones, Fan, Franchini, Schartl, & Meyer, 2013), pyrad can be equally as effective and generally discovers more loci than stacks (Pante et al., 2015). For example, Mort et al. (2015) utilized the pyrad pipeline to generate a well‐resolved phylogeny of a plant genus.

In pyrad, adaptor‐trimmed sequences with quality scores were used for demultiplexing. As a quality control measure, reads were kept if there were fewer than 15 sites that had Phred scores ≤20. The minimum number of reads for cluster formation and putative loci identification was set to seven. Putative pyrad loci were retained if at least ten individuals shared a locus. If we required more individuals to share a locus, we lost a considerable number of loci (see Results); therefore, we kept the minimum number of individuals (i.e., samples in pyrad) to ten. Up to 100 SNPs per locus (default for parameter ## 26.opt) were allowed. Using pyRAD generated data matrices, maximum likelihood trees with 10,000 ultrafast bootstrap replicates (Minh, Nguyen, & Haeseler, 2013) were inferred using iq‐tree (Nguyen, Schmidt, Haeseler, & Minh, 2015). The model used for both species was GTR + I + G4. Tree files were visualized using figtree (Rambaut, 2007).

## 3 RESULTS

A cumulative total of approximately 120 million raw reads were generated from 151 individuals across ten *N. menapia* and seven *N. terlooii* sampling sites in AZ. All of the *N. terlooii* individuals were retained after filtering and 82 of the 88 *N. menapia* were retained. After filtering, up to 10,740 were loci were recovered per sampling site, depending on the method and species (Table 2). Each locus contained one SNP for stacks and up to 100 SNPs for PYRAD.

**Table 2:** A comparison of the number of loci discovered for either *Neophasia* spp. with the pyrad and stacks pipelines

Species	Number of loci per pipeline	
	pyrad	stacks
*Neophasia menapia*	10,740	4,732
*Neophasia terlooii*	9,351	3,125

The same individuals were included in each pipeline.

### 3.1 Quantifying genetic variability and spatial relationships

**Figure 2:**
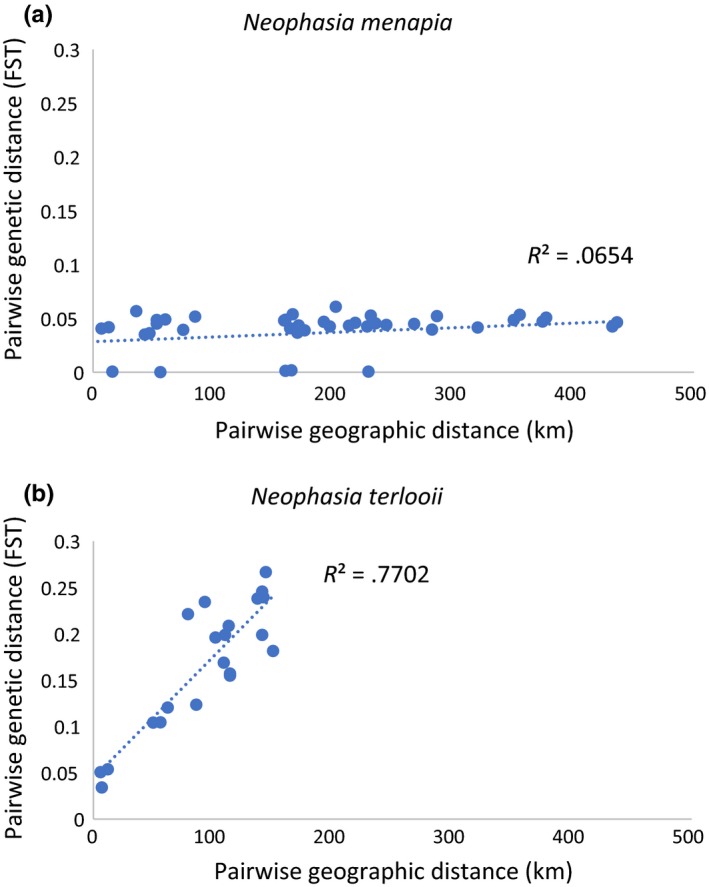
Plot of genetic distance (*F*_ST_) as a function of geographic distance for (a) *Neophasia menapia* and (b) *Neophasia terlooii*. Each point represents a pairwise comparison of two individuals from different collection sites

Within the filtered individuals, 90% of *N. menapia* loci and 70.8% of *N. terlooii* loci were in Hardy–Weinberg equilibrium. Heterozygosity estimates were greater within *N. terlooii* sampling locations than within *N. menapia* sampling locations (Table 3). Fixation indices between *N. terlooii* sites were, on average, five times greater than those between *N. menapia* sites: *N. menapia* site pairs ranged from 7.31e^−5^ to 0.061, while *N. terlooii* site pairs ranged from 0.034 to 0.267 (Table S1). There were significant amounts of variation between mountains and between sampling sites within mountains for both *N. menapia* and *N. terlooii*, and there was a significant amount of variation within *N. terlooii* individuals as indicated by the analysis of molecular variance (Table 4). When plotting genetic distance as a function of geographic distance, there was a positive correlation between the two for *N. terlooii* (Figure 2b). This was evident with significant isolation by distance (*r* = .9062, *p* = .001,) from a Mantel test. *Neophasia menapia* did not show significant isolation by distance (*r* = −.081, *p* = .583), nor did genetic distance correlate with geographic distance (Figure 2a).

**Table 3:** Heterozygosity estimates for each geographic sampling site

Species	Site	Heterozygosity estimate	Species	Site	Heterozygosity estimate
*Neophasia menapia*	BL	0.1316077	*Neophasia terlooii*	CH	0.16146579
	FR	0.1272531		CR	0.14976759
	GN	0.1297881		GM	0.09822872
	HW	0.1306090		HC	0.16208089
	LO	0.1276648		LM	0.12387499
	MO	0.1307747		MD	0.14302912
	NJ	0.1318904		SW	0.16317188
	PT	0.1224726			
	SC	0.1326815			
	SJ	0.1336801			

**Table 4:** AMOVA for each species of *Neophasia*. Some mountains contain multiple collection sites

Comparison	Df	Sum sq	Mean sq	Sigma	% Covariance	*p*‐Value
*Neophasia menapia*						
Within samples	4	475.7429	118.93572	0.5333659	0.5519891	.2524
Between samples within site	5	511.4623	102.29247	0.4818000	0.4986227	.4676
Between site within mountain	64	6,132.8688	95.82607	0.2150782	0.2225879	.0146*
Between mountain	74	7,059.2980	95.39592	95.3959184	98.7268002	.0311*
Total	147	14,179.3720	96.45831	96.6261625	100.0000000	
*Neophasia terlooii*						
Within samples	65	4,622.5000	71.11538	71.11538462	73.2834232	<.0001***
Between samples within site	58	4,114.6847	70.94284	−0.08627229	−0.0889024	.5137
Between site within mountain	2	205.2079	102.60394	1.76621812	1.8200634	.0015**
Between mountain	4	2,755.1536	688.78839	24.24623981	24.9854158	.0001***
Total	129	11,697.5462	90.67865	97.04157026	100.0000000	

Note

Asterisks denote statistical significance.

### 3.2 Identifying population clusters

**Figure 3:**
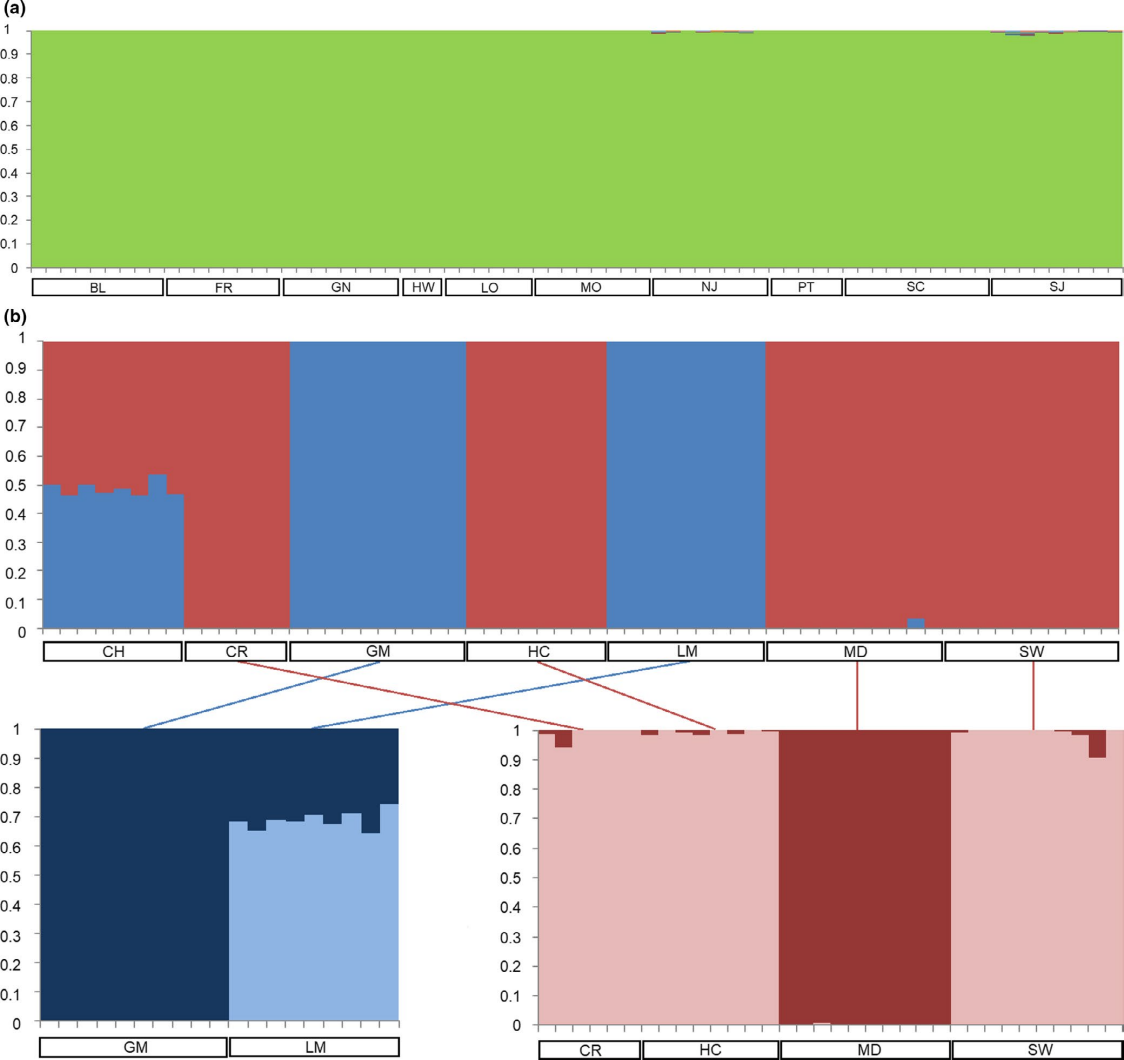
structure clustering for (a) *Neophasia menapia* and (b) *Neophasia terlooii*. *K* = 9 is plotted for *N. menapia*. Substructure is shown in *N. terlooii* in which the cluster containing CR, HC, MD, and SW can be broken down to two clusters, one with MD and the other with CR, HC, and SW. Individuals are grouped by sampling site, indicated by the open boxes beneath each diagram

For *N. terlooii*, Bayesian hierarchical clustering using structure suggested an optimum *K* of 2 based on delta *K* (Evanno, Regnaut, & Goudet, 2005) and the mean log probabilities (Table 5). Substructure was evident in one of the primary clusters in which the site in the Santa Rita Mountains separated from the three sites in the Huachuca Mountains An optimal *K* for *N. menapia* was not clear using these methods with delta *K* suggesting a *K* = 9, but mean log probabilities increasing with *K* and the highest probability being for the maximum *K* tested: *K* = 15 (Table 5). Assignment probability plots of individuals to each of several population clustering options are presented in Figure 3.

**Table 5:** Bayesian clustering results for possible numbers of clusters of individuals (*K*)

Species	*K*	Reps	Mean LnP(*K*)	Stdev LnP(*K*)	Ln′(*K*)	|Ln″(*K*)|	Delta *K*
*Neophasia menapia*	1	20	−157,726.23	5.28	NA	NA	NA
	2	20	−157,737.73	6.31	−11.50	13.63	2.16
	3	20	−157,735.60	4.97	2.13	16.23	3.27
	4	20	−157,717.24	7.34	18.36	3.83	0.52
	5	20	−157,702.71	3.63	14.53	12.10	3.33
	6	20	−157,700.28	4.95	2.43	0.20	0.04
	7	20	−157,697.65	3.26	2.63	7.07	2.17
	8	20	−157,687.95	4.19	9.70	13.29	3.17
	9	20	−157,691.54	2.28	−3.59	9.57	**4.20**
	10	20	−157,685.57	4.61	5.98	5.67	1.23
	11	20	−157,685.26	7.88	0.31	0.65	0.08
	12	20	−157,684.30	3.05	0.96	3.95	1.29
	13	20	−157,679.39	3.40	4.91	8.08	2.37
	14	20	−157,682.56	5.11	−3.17	6.74	1.32
	15	20	**−157,678.99**	2.76	3.57	NA	NA
*Neophasia terlooii*	1	20	−138,566.38	0.77	NA	NA	NA
	2	20	**−123,727.67**	9.46	14,838.72	25,799.73	**2,725.93**
	3	20	−134,688.68	255.10	−10,961.02	11,796.17	46.24
	4	20	−133,853.53	718.25	835.15	61.90	0.09
	5	20	−133,080.28	318.51	773.26	1,267.90	3.98
	6	20	−133,574.92	179.67	−494.64	647.31	3.60
	7	20	−133,422.25	494.84	152.67	2,053.04	4.15
	8	20	−135,322.63	1,196.98	−1,900.38	3,744.06	3.13
	9	20	−133,478.94	36.89	1,843.69	3,222.10	87.34
	10	20	−134,857.35	1,271.75	−1,378.41	1,063.56	0.84
	11	20	−135,172.20	715.26	−314.85	53.01	0.07
	12	20	−135,434.04	295.09	−261.84	963.96	3.27
	13	20	−136,659.84	129.87	−1,225.80	4,117.10	31.70
	14	20	−133,768.55	491.69	2,891.30	4,571.78	9.30
	15	20	−135,449.03	1,248.08	−1,680.49	NA	NA

Note

Bold values indicate the most likely option(s) for *K*.

### 3.3 Phylogenetic analysis

**Figure 4:**
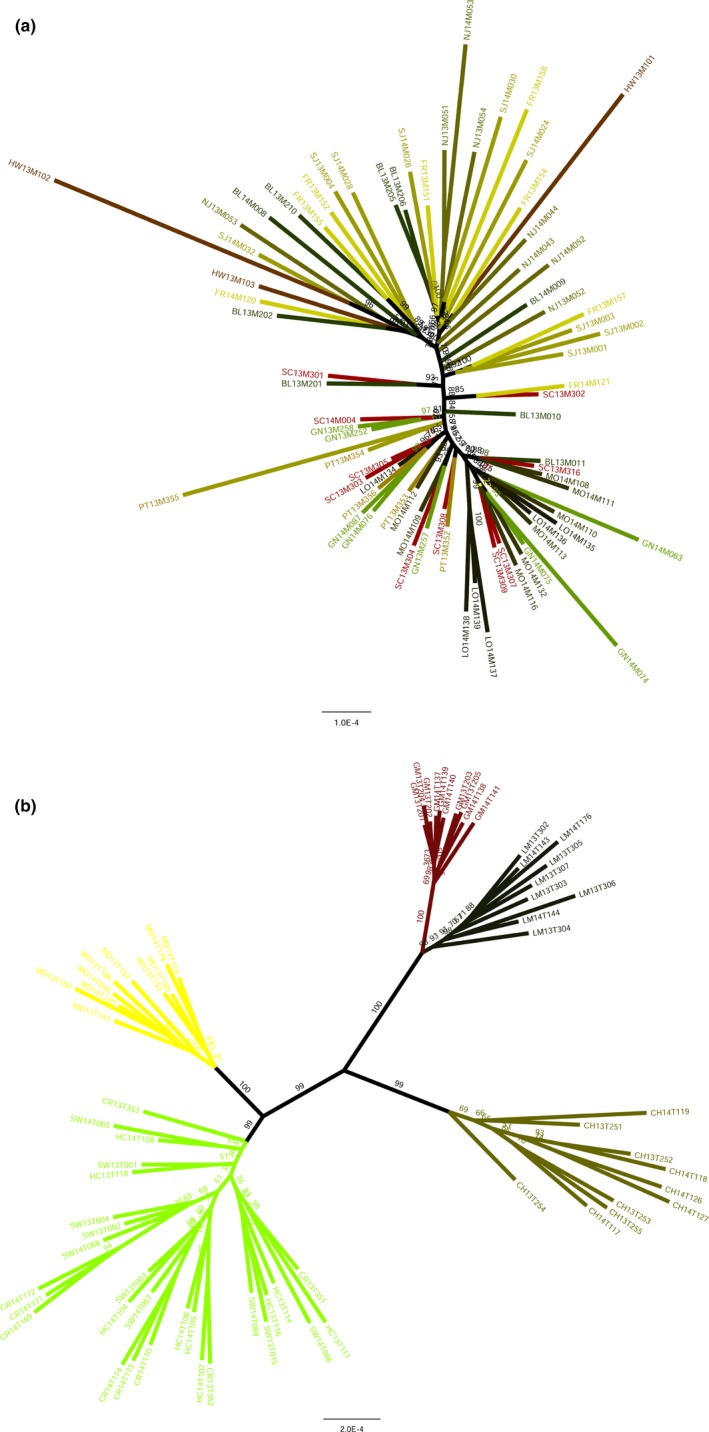
Maximum likelihood trees from ddRADSeq data for *Neophasia* spp. from Arizona. Each tip represents an individual color coded to its sampling site. Tip labels refer to sampling site abbreviation, year collected, and specimen number. Branch labels indicate support from 10,000 ultrafast bootstrap replicates. (a) Tree for *Neophasia menapia*, (b) tree for *Neophasia terlooii*

pyrad allowed us to use the ddRADSeq data for inferring phylogenetic relationships among individuals across our sampling sites. Specifying that 10% of individuals must share a locus produced large matrices with a lot of missing data in the analysis. However, using a more stringent threshold, such as requiring a minimum of 80% of individuals to share a locus (as used in the stacks populations pipeline for genotyping) resulted in unresolved trees with low branch support. Despite the risk of greater missing data, which can negatively affect accuracy in smaller datasets (Lemmon, Brown, Stanger‐Hall, & Lemmon, 2009), gathering a greater number of loci and generating larger supermatrices results in more fully resolved phylogenies (Wagner et al., 2013). Unrooted trees were used to depict the relationships between individuals. *Neophasia menapia* formed a polytomy, but there were a few well‐supported clades of up to three individuals from the same sampling sites (Figure 4a). Individuals of *N. terlooii* formed well‐supported clades corresponding to each mountain range (Figure 4b). Individuals from sites within the Huachuca Mountains were polyphyletic.

## 4 DISCUSSION

### 4.1 Overview

The sky islands are considered a neglected biodiversity hotspot (Felger & Wilson, 1994). Research on the biodiversity of the sky islands has focused primarily on dispersal and vicariance events associated with the Pleistocene epoch with subsequent divergence associated with biotic (Carstens & Knowles, 2007; Tennessen & Zamudio, 2008) and abiotic factors (Smith & Farrell, 2005). Within the sky islands, geographic structuring in genetic relationships has been observed in flightless beetles (Ober et al., 2011; Smith & Farrell, 2005), an ant (Favé et al., 2015), and a jumping spider (Masta, 2000). Our work here demonstrates that, despite their flight capability, *N. terlooii* has highly structured genetic patterns, which may be the consequence of abiotic factors, such as climate change and spatial habitat connectivity. The AZ sky islands and surrounding areas occupied by *Neophasia* are natural landscape‐scale laboratories, providing comparisons for how connectivity influences the evolution of organisms in fragmented habitats.

Our results support the hypothesis that habitat connectivity affects the genetic structure of *Neophasia*. Our geographic sampling strategy facilitated population genetic comparisons between individuals in large, contiguous patches and those in small, isolated patches. We were also able to compare individuals between clusters of proximal and distant sampling sites within single patches. Nearly all evidence suggests that *N. menapia* is panmictic and there is minimal genetic structure with respect to sampling sites, most of which are connected by a largely contiguous habitat belt. There is strong evidence for genetic structure within *N. terlooii* as populations of butterflies on different sky islands are distinct from each other. Despite the dispersal abilities of *Neophasia* butterflies, present day desert and grassland matrices between coniferous forests limit their dispersal.

### 4.2 *Neophasia menapia* is panmictic in AZ

Traditionally, population genetic studies required at least tens of individuals per sampling site to make accurate inferences. Willing, Dreyer, and Oosterhout (2012) found that the original Wright (1951) *F*_ST_ overestimated differentiation with small sample sizes (*n* ≤ 6), but sample sizes from individual populations can be small (*n* = 6) provided there is a large number of genetic markers (*k* > 1,000). In our study, at least 3,000 SNPs were genotyped using stacks, and thus our sample sizes, averaging ten individuals per site, were appropriate for making *F*_ST_ comparisons.

Of the 36 pairwise *F*_ST_ indices for *N. menapia*, 28 were <0.05, suggesting panmixia. The AMOVA indicated that there was little, but significant genetic variation between sites for *N. menapia*, but additional analyses suggested this variation is not sufficient to generate robust population or phylogenetic inferences. The two *N. menapia* sites that were most distant geographically from each other (NJ and FR at 436 km) had an *F*_ST_ value of 0.046, but the two closest *N. terlooii* sites (HC and SW at 7.6 km) had a similar *F*_ST_ of 0.034 (compare Figure 1 and Table S1). Most other *N. terlooii* population pairs on different mountains had *F*_ST_ indices of approximately 0.20. This suggests that, despite the greater geographic distance between several *N. menapia* sampling sites, gene flow is occurring or has occurred more frequently between *N. menapia* sites than between *N. terlooii* populations.

The sampling sites for *N. menapia* were all largely on one habitat island. However, the Grand Canyon geographically isolated the sites NJ and SJ on the Kaibab Plateau from the rest of the sites to the south of the canyon. The connectivity of habitat is likely responsible for the apparent panmixia of *N. menapia*, as ponderosa pine forest is largely contiguous and encompasses all sampling sites south of the Grand Canyon. The ddRADSeq phylogeny was essentially a polytomy (Figure 4a). There is some evidence for the populations north of the Grand Canyon to be distinct from those south of the canyon. This is apparent in the structure plot (Figure 3b). However, the structure analysis did not produce a clear, definitive *K*, which appears to provide further evidence for panmixia. We conclude that *N. menapia* is most likely panmictic in AZ and flight across the Grand Canyon is possible. However, given the possibility that some ancestral polymorphism was retained, the Grand Canyon may restrict gene flow to some degree given the lack of host plants at the lower elevations across the canyon.

Isolation by distance (IBD; Wright, 1943) occurs when genetic differentiation is due to limited gene flow across long distances rather than biologically relevant barriers in the environment. Populations arising from dispersal events tend to be geographically clustered and represent some pattern of spatial autocorrelation (Meirmans, 2012). Mantel tests cannot differentiate between patterns arising from such clustering and those from IBD (Meirmans, Goudet, & Gaggiotti, 2011). The latter authors argue that it is best to use a clustering method that accounts for the geographical locations of the samples. The Mantel test suggested that IBD was not significant between *N. menapia* sampling sites, which also did not show any consistent or well‐supported clustering. This makes sense because there were roughly equivalent *F*_ST_ indices between proximal and distant sites (Figure 2a). Because we ran the Mantel test without specifying the geographical location data and there was evidence for hierarchical clustering within *N. terlooii* sampling sites, it is not surprising that the Mantel test was significant for *N. terlooii*. We conclude that the isolation by distance within *N. terlooii* is more likely due to hierarchical clustering on isolated mountains.

### 4.3 Sky islands are responsible for *Neophasia terlooii* population structure

During our surveys, *Neophasia* butterflies were only observed at elevations between 1,700 and 2,600 m above sea level, and these elevations contain the host trees. The lowering of forest ecotones during the last glacial maxima (Thompson & Anderson, 2000) would have had little impact with respect to changes in past connectivity of our *N. menapia* sampling sites, but it would have connected several *N. terlooii* populations in the sky islands (Figure 1). The *N. terlooii* population in the Santa Rita Mountains (MD) would have been connected with the population in the Huachuca Mountains (sites HC, SW, and CR) and this largely agrees with the molecular data, as it is clustered with the latter three in the first structure analysis. The population in the Pinaleño Mountains (GM) would have been connected with the population in the Chiricahua Mountains (CH), and this is evidenced by admixture with the former. The Santa Catalina Mountains (LM) is the only range that would not have been connected, yet the molecular data suggest admixture with CH and membership to a cluster also containing GM.

*Neophasia terlooii* populations show a very clear hierarchical population structure with respect to populations on each sky island. The northwest–southeast oriented topographic grain of the sky islands (see Felger & Wilson, 1994) could explain their north–south‐nested hierarchy. Similarly, the ant *Monomorium emersoni* Gregg exhibited north and south groupings in the sky islands (Favé et al., 2015). *Neophasia terlooii* populations LM and GM are on the two northern‐most sky islands and are distinct from the remaining southern populations (Figure 3b). At the next level in the hierarchical structure within the remaining southern clusters, the population in the Santa Rita Mountains (MD) is separated into its own cluster. As discussed above, MD would have been isolated historically. The sites within the Huachuca Mountains (HC, SW, and CR) form the final population with respect to mountain. Given the admixture between northern and southern clusters seen in CH, this range may have been a stepping stone between the two.

The panmixia of *N. terlooii* within the Huachuca Mountains is reflected in the ddRADSeq phylogeny where these sites are polyphyletic (Figure 4b) and in the structure plot where these sites are all assigned to one cluster (Figure 3b). The optimum *K* of 2 found using structure reflects the uppermost level in the aforementioned nested hierarchy, but with some admixture between clusters for the CH population. Based on the structure analysis and phylogeny, four populations are most likely.

### 4.4 Dispersal limitations and biogeographical considerations for *Neophasia*

It is evident that the geographic distance between the sky island mountains is enough to isolate *N. terlooii* populations. The shortest distance between any two sky islands in our study is 55 km (between the Santa Rita and Huachuca Mountains, populations MD and HC + SW + CR, respectively). From the Kaibab Plateau to the south rim, the Grand Canyon spans roughly 13 km of unsuitable habitat for *N. menapia* and there not strong evidence to suggest this distance inhibits gene flow. At their closest point, the northern‐most sky island and ponderosa pine habitat in the southern White Mountains are roughly 70 km apart. This distance appears sufficient to maintain allopatry between *N. menapia* and *N. terlooii*, but geographic distance may not be the only factor.

Even if the geographic distance is within the dispersal capabilities of an organism, or if the habitat between populations is suitable, allopatry can persist for biological reasons such as mating incompatibility, competition, or predation (Tennessen & Zamudio, 2008). The female *N. terlooii* has long been suspected a mimic of the monarch butterfly (Poulton, 1914), and, as discussed in Halbritter, Gordon, Keacher, Avery, and Daniels (2018), mimicry may be advantageous to *N. terlooii* while *N. menapia* may be vulnerable to predation if it were to venture into the range of *N. terlooii*. To date, *N. menapia* has not been collected or seen in the sky islands and *N. terlooii* has not been collected or seen north of the sky islands. Crossing the barrier between the sky islands and the White Mountains is likely a rare event, and any transplants are incapable of starting new populations for reasons that are beyond the scope of this paper. Further sampling of the genetic diversity of both species of *Neophasia* throughout their respective ranges would provide the data necessary to unravel their biogeographic history in western North America and would contribute to our work here and to knowledge of the evolution of biodiversity in the sky islands. Additionally, the timing and direction of dispersal events is key to further exploring their biogeography. This remains for future study and will require more accurate methods of divergence time estimation at the population level.

## 5 CONCLUSIONS

We extracted genome‐wide data from 151 individuals and used this data to infer population structure and generate phylogenies for a nonmodel taxon in a neglected biodiversity hotspot. Despite differences in the number and type of loci discovered across the genomic pipelines used, we reached congruent biological conclusions regarding *Neophasia* population structure in AZ. As our ability to analyze genomic data catches up with our ability to generate data, complex biogeographical studies using nonmodel taxa at very shallow phylogenetic levels will be increasingly common and affordable. With improved algorithms and faster processors, total‐evidence approaches are becoming increasingly feasible, especially for projects with minimal or short‐term funding.

## CONFLICT OF INTEREST

None declared.

## AUTHOR CONTRIBUTIONS

D.A.H. led this project as a part of his dissertation research; D.A.H., A.Y.K., and J.C.D. conceived the ideas; D.A.H. collected the data; D.A.H. and C.G.S. analyzed the data; and D.A.H. led the writing.

## Data Availability

Genetic data, code, alignments, and other output files: Dryad https://doi.org/10.5061/dryad.4mw6m906h
