# Supplementary material for: Phylogeography and population genetics of pine butterflies: Sky islands increase genetic divergence
Source: Ecol Evol. 2019 Nov 7;9(23):13389–401. doi: 10.1002/ece3.5793 (PMC6912906; doi:10.1002/ece3.5793)
Supplement: Supplementary file 1 [file ECE3-9-13389-s001.docx]

**Table S1.** Comparisons of F_ST_ values between sampling sites for each *Neophasia* species. NJ and SJ are on the Kaibab Plateau, BL, SC, LO, HW, and MO are on the Mogollon Rim, and FR, GN, and PT are in the White Mountains. CH, SW, and CR are in the Huachuca Mountains, MD is in the Santa Rita Mountains, CH is in the Chiricahua Mountains, LM is in the Santa Catalina Mountains, and GM is in the Pinaleño Mountains. Asterisks indicate significant difference from 0.

| Sites for *Neophasia menapia* | | | | | | | | | |
| --- | --- | --- | --- | --- | --- | --- | --- | --- | --- |
|  | BL | FR | GN | HW | LO | MO | NJ | PT | SC |
| FR | 0.041451* |  |  |  |  |  |  |  |  |
| GN | 0.04494* | 0.049018* |  |  |  |  |  |  |  |
| HW | 7.31E-05 | -0.00294 | 0.000518 |  |  |  |  |  |  |
| LO | 0.045159* | 0.052286* | 0.052738* | -0.00304 |  |  |  |  |  |
| MO | 0.03949* | 0.043991* | 0.04686* | -0.00015 | 0.048469* |  |  |  |  |
| NJ | 0.043487* | 0.046427* | 0.050667* | 0.001845 | 0.053864* | 0.046014* |  |  |  |
| PT | 0.045398* | 0.051792* | 0.056822* | -0.00835 | 0.060777* | 0.047977* | 0.053425* |  |  |
| SC | 0.036251* | 0.039626* | 0.042474* | 0.000553 | 0.041721* | 0.035113* | 0.038805* | 0.042519* |  |
| SJ | 0.04074* | 0.042569* | 0.047113* | 0.001293 | 0.048884* | 0.043208* | 0.04053* | 0.048485* | 0.036828* |

| Sites for *Neophasia terlooii* | | | | | | |
| --- | --- | --- | --- | --- | --- | --- |
|  | CH | CR | GM | HC | LM | MD |
| CR | 0.169031* |  |  |  |  |  |
| GM | 0.234355* | 0.245719* |  |  |  |  |
| HC | 0.154838* | 0.053884* | 0.238089* |  |  |  |
| LM | 0.198784* | 0.208792* | 0.123628* | 0.196207* |  |  |
| MD | 0.181537* | 0.120329* | 0.266571* | 0.10424* | 0.221295* |  |
| SW | 0.157264* | 0.050592* | 0.23953* | 0.034166* | 0.198975* | 0.104485* |
