# Supplementary material for: Phylogeography and population genetics of pine butterflies: Sky islands increase genetic divergence
Source: Ecol Evol. 2019 Nov 7;9(23):13389–401. doi: 10.1002/ece3.5793 (PMC6912906; doi:10.1002/ece3.5793)
Supplement: Supplementary file 2 [file ECE3-9-13389-s002.docx]

SUPPLEMENTAL DOCUMENT 1

DNA EXTRACTION AND LIBRARY ASSEMBLY PROTOCOLS

DNA Extraction: OmniPrep

1. Ten specimens were selected for extraction
2. Specimen identifications were written on the lids of 10 1.5-mL autoclaved microtubes
3. Each tube was filled with 500 µL of Genomic Lysis Buffer
4. Thoracic tissue was dissected from each specimen and placed into the buffer in its respective tube
5. The 10 tissue samples were each homogenized with clean microcentrifuge pestles
6. Five µL of Proteinase K was added to each tube and tubes were mixed by vortexing for 5 sec then centrifuged in a mini centrifuge (~6,000 rpm) for 5 sec
7. Tubes were incubated for 3 h at 56° C, then vortexed for 5 sec and mini-centrifuged for 5 sec. This was repeated every hour
8. Specimen identifications were written on the lids 20 1.5-mL autoclaved microtubes
9. Tubes were cooled by placing them in a room-temp tube rack. 200 µL of chloroform were added to each tube and mixed by inverting 10 times. Tubes were centrifuged at 13,200 rpm for 11 min and the upper phase was carefully transferred to new 1.5-µL microtubes
10. Fifty µL of DNA Stripping Solution was added to each tube and mixed by inverting 10 times. Tubes were incubated at 56° C for 10 min
11. Eighty % ethanol, 2-propanol, and Mussel glycogen were chilled in an bucket
12. Tubes were cooled by placing them in a room-temp tube rack. 100 µL of Precipitation Solution were added to each tube and mixed by inverting 10 times. A cloudy white precipitate was observed
13. Tubes were centrifuged at 13,200 rpm for 7-8 min. The clear supernatant was quickly transferred to clean 1.5-µL tubes
14. Two µL of Mussel glycogen were added to each tube and mixed by inverting 10 times
15. Five hundred µL of 2-propanol were added to each tube and mixed by inverting 10 times
16. Tubes sat on ice for 30 min
17. Tubes were centrifuged at 13,200 for 11 min and then the supernatant was carefully discarded by pouring it into a clean Kim Wipe. A DNA pellet was seen in the bottom of each tube
18. Seven hundred µL of 80% ethanol were added to each tube by gently squirting it onto the inside walls of the tubes. Tubes were mixed by inverting 10 times to wash the DNA pellets
19. Tubes were centrifuged at 13,200 rpm for 1.5 min. The ethanol wash was carefully poured into a clean Kim Wipe, ensuring that the pellets remained adhered to their tubes. Tubes were allowed to dry but the care was taken to ensure the pellets did not dry
20. Fifty µL TE Buffer were added to each tube to dissolve the pellets. Tubes were incubated at 56° C for 45 min and then cooled to room temp before storing in a -20° C freezer
21. One µL of RNase was added to each tube before RADSeq library prep

Library Assembly: ddRADSeq

This ddRADSeq protocol is a variation of the Peterson et al. (2012) protocol and it utilized by the McDaniel Lab at the University of Florida. Stuart McDaniel and Adam Payton have written this revised the protocol and we have made further revisions tailored to this project.

Primary reagents:

- EcoRI-HF (NEB, R3101 20,000 units/mL)
- MseI (NEB, R0525 10,000 units/ mL)
- T4-DNA ligase (NEB, M0202 400,000 units/ mL)
- ATP (NEB, P0756 10mM)
- dNTP (NEB, N0447 8um each nucleotide)
- Q5 high fidelity DNA polymerase (NEB, M0491 2,000 units/ mL)
- OneTaq standard PCR mastermix (NEB, M0486)

Adaptor Hybridization

Individual complementary single-stranded oligonucleotides were hybridized to create double-stranded adaptors, which contained a "sticky-end" overhanging enzyme cut-site sequence, the associated barcode (either 8, 9, 10, or 14 bp), an Illumina flowcell priming site, and a sequencing primer. The hybridization process was performed in a high-salt environment utilizing a thermocycler.

10x hybridization buffer = 100mM Tris-HCl pH 8 + 500mM NaCl + 10mM EDTA + H_2_O

For 100 mL:

- 10 mL 1M Tris-HCl pH8
- 10 mL 5M NaCl
- 0.3722 g EDTA
- 80 mL Filtered H_2_O

The following were mixed to create a 10 µM stock of hybridized (double-stranded) adaptor:

- 10 µL Adaptor_1 (100µm)
- 10 µL Adaptor_1.b (100µm)
- 10 µL 10x Annealing Buffer
- 70 µL H_2_O

This was repeated for all adaptors (i.e., on all 5’ and 3’ adaptor pairs for EcoRI and MseI), each were mixed well with a plate vortexer, placed into a thermocycler, incubated at 97.5° C for 2.5 min, and then cooled at a rate ≤ 3° C/min until the solutions reached 21° C. After completion, the thermocycler was held at 4° C until adaptors were moved to a freezer.

Enzyme Digestion

Genomic DNA concentrations ranged from 10 ng/µL to 900 ng/µL but most samples were in the range of 100-400 ng/µL. Those that were particularly low (based on a later PCR quality control checkpoint) were doubled at a later step (see below) to normalize the quantity of DNA for each sample.

1. Six µL of each sample’s genomic DNA were placed into a well of a 96-well plate, which was kept over ice
2. Master Mix 1 was prepared for 204 total reactions (130% of 157 samples to account for reagent loss to pipette adhesion)

For each reaction:

- 0.9 µL Cutsmart 10x buffer
- 0.28 µL EcoRI-HF enzyme
- 0.12 µL MseI enzyme
- 1.7 µL H_2_O

1. Master Mix 1 was mixed well with a plate vortexer and otherwise kept on ice
2. Three µL of Master Mix 1 were added to each DNA sample to achieve 9 µL total volume in each well
3. The 96-well plate was sealed with adhesive foil, mixed well with a plate vortexer, centrifuged, and incubated at 37° C for 8 h on a thermocycler with a heated lid set to 50° C

Adaptor Ligation

The double-stranded EcoRI and MseI adaptors were thawed and all steps below were carried out on ice.

1. One µL of the diluted EcoRI adaptor was added to each sample of digested DNA

2. Master Mix 2 was prepared at 190 X

For each reaction:

- 0.4 µL Cutsmart buffer 10x
- 1.3 µL ATP 10 µm
- 0.2 µL T4 Ligase enzyme
- 0.1 µL H_2_O
- 1.0 µL MseI adaptor

3. Three µL of Master Mix 2 were added to each digested DNA sample to yield a total reaction volume of 14 µL per well

4. The 96-well plate was sealed with adhesive foil, mixed well with a plate vortexer, centrifuged, and incubated at 16° C for 6 h on a thermocycler with a heated lid set to 50° C. Samples were then stored in a freezer until the next step.

PCR 1

This PCR was ran to ensure that library constructions were successful, i.e., that both adaptors were ligated to their respective fragment ends. step will be used to test the success of library construction of each individual sample. PCR products at this step will not be used in any downstream steps.

Master Mix 3 was prepared at 170 X

For each reaction:

- 8 µL NEB One-Taq 2x Master Mix
- 0.8 µL 10 µm F and R primers for Illumina (premixed)
- 6.2 µL H_2_O

1. Fifteen µL of Master Mix 3 was added to each well in two 96-well plates to fill 157 wells. Then 1 µL of each sample's DNA restriction/ligation product was added to a well.

2. The 96-well plates were sealed with adhesive foil, mixed well with a plate vortexer, centrifuged, and placed into a thermocycler with a heated lid (50° C) under the following conditions: 94° C for 2 min then 20 cycles of (94° C for 30 sec, 60° C for 30sec, 68° C for 45° sec). After completion, the thermocycler was held at 6° C.

Agarose Gel 1

The PCR 1 products for each sample were ran on a 1.4 % agarose gel with a 100-bp ladder. All products were successfully amplified and fragments ranged from ~50 bp to ~700 bp.

Size Selection

Three µL of each sample, except 6 µL of samples that produced faint bands in PCR 1, were all combined into a 1.5 mL tube. The tube was delivered to the University of Florida's ICBR genomics facility where they concentrated the DNA to be fractioned by fragment sizes using Pippin ELF.

PCR 2

PCR 2 was run on each of the size-selected fractions to aid in selecting the fraction with the highest concentration of DNA. The same procedures were followed as in PCR 1, except it was only replicated 8 X. Based on the gel, the fractions of 330-475 bp and 275-380 bp had the brightest bands and therefore the highest concentrations.

PCR 3

PCR 3 was run to ligate the flowcell binding sequence to the barcoded DNA fragments, which included the size-selected fractions of 330-475 bp and 275-380 bp.

Master Mix 4 was prepared at 36X. This ensured that each fraction was replicated 16 times plus 4 extra replicates to account for adhesion to pipette tips.

For each reaction:

- 3 µL Q5 buffer 5x
- 0.3 µL DNTP
- 1 µL Primers F&R combined 5 µM
- 0.15 µL NEB high-fidelity Q5 Taq
- 9.55 H_2_0

Each well containing 14 µL of Master Mix 4 had 1 µL of size-selected DNA from each fraction added. The 96-well plates were sealed with adhesive foil, mixed well with a plate vortexer, centrifuged, and placed into a thermocycler with a heated lid (50° C) under the following conditions: 98° C for 30 sec, 10 cycles of (98° C for 15 sec, 60° C for 30 sec, 72° C for 30 sec), and holding 4-10° C when completed.

Agarose Gel 2

Two µL of the products of PCR 3 were loaded into 1.4 % agarose gel with a 100-bp ladder. All products produced a band in the target region and were therefore successful.

Sequencing

All PCR 3 reactions were pooled into a single 1.5 µL tube. I used a QIAquick PCR Purification Kit to remove enzymes and buffers. The tube was then delivered to the University of Florida's ICBR genomics facility where they used AMPure to remove primers. I specified the library size of 275-475 bp per library, which included the 100-bp primers. I used a PhiX spike at 10% and a cluster target density of 160K. Final reads were uploaded to Illumina's BaseSpace server.
